# A disclosure form for work submitted to medical journals – a proposal from the International Committee of Medical Journal Editors

**DOI:** 10.2471/BLT.20.252353

**Published:** 2020-03-01

**Authors:** Darren B. Taichman, Joyce Backus, Christopher Baethge, Howard Bauchner, Annette Flanagin, Fernando Florenzano, Frank A. Frizelle, Fiona Godlee, Laragh Gollogly, Abraham Haileamlak, Sung-Tae Hong, Richard Horton, Astrid James, Christine Laine, Pamela W. Miller, Anja Pinborg, Eric J. Rubin, Peush Sahni

**Affiliations:** a Annals of Internal Medicine, 190 N. Independence Mall W., Philadelphia, PA 19106—1572, United States of America (USA).; b National Library of Medicine, Bethesda, USA.; c Deutsches Ärzteblatt (German Medical Journal), Köln, Germany.; d JAMA (Journal of the American Medical Association), Chicago, USA.; e Revista Médica de Chile (Medical Journal of Chile), Santiago, Chile.; f New Zealand Medical Journal, Wellington, New Zealand.; g The BMJ (British Medical Journal), London, England.; h Science Division, World Health Organization Geneva, Switzerland.; i Ethiopian Journal of Health Sciences, Jimma, Ethiopia.; j Journal of Korean Medical Science, Seoul, Republic of Korea.; k The Lancet, London, England.; l Annals of Internal Medicine, Philadelphia, USA.; m New England Journal of Medicine, Waltham, USA.; n Ugeskrift for Laeger (Danish Medical Journal), Copenhagen, Denmark.; o All India Institute of Medical Sciences, Delhi, India.

Many factors, including professional and personal relationships and activities, can influence the design, conduct, and reporting of the clinical science that informs health care decision. The potential for conflict of interest exists when these relationships and activities may bias judgement.^1^ Many stakeholders—editors, peer reviewers, clinicians, educators, policymakers, patients, and the public—rely on the disclosure of authors’ relationships and activities to inform their assessments. Trust in the transparency, consistency, and completeness of these disclosures is essential.

Ten years ago, the International Committee of Medical Journal Editors (ICMJE) adopted the “ICMJE Form for the Disclosure of Potential Conflicts of Interest” as a uniform mechanism for collecting and reporting authors’ relationships and activities that readers might consider relevant to a published work.^2^

The goal was to avoid the confusion (and often ensuing controversy) created when journals vary in how they collect and report this information. We believe a uniform disclosure form has been helpful, but problems remain. First, the software supporting the current form is increasingly problematic, making its use difficult or impossible for an increasing number of authors. More important, however, is that many authors and readers misunderstand, misapply, or misinterpret the disclosures.

Although some individuals violate the public trust by purposefully hiding relevant relationships and activities, we believe most authors are committed to transparent reporting and consider it as vital to the advancement of clinical science. Nonetheless, disagreement, confusion, and controversy regarding authors’ disclosures arise when opinions differ over which relationships and activities to report. An author might not report an item that others deem important because of a difference in opinion regarding what is “relevant,” confusion over definitions, or a simple oversight. Some authors may be concerned that readers will interpret the listing of any item as a “potential conflict of interest” as indicative of problematic influence and wrongdoing, a concern often raised regarding the requirement to report publicly funded grants. For their part, some readers fail to recognize that their own relationships and activities influence how they assess the work of others and what they deem to be a “conflict” for others or themselves.

We propose several changes to the ICMJE disclosure form to help address these issues. First, words matter. Despite including the word “potential,” a form entitled “…for the Disclosure of Potential Conflicts of Interest” may imply that any relationship or activity listed represents a problematic influence or wrongdoing. The proposed new title, “The ICMJE Disclosure Form,” aims to dispel that interpretation and potential stigma. Second, we no longer ask authors to decide what might be interpreted as a potential conflict of interest. Authors disclose their relationships and activities so that readers can decide whether these relationships or activities should influence their assessments of the work. Further, to avoid omissions–inadvertent or purposeful–we now provide a checklist of relationships and activities for authors to complete.

We welcome feedback about the proposed new form, which is available with a link to provide comments, at www.icmje.org. We will consider comments received by 30 April 2020, before finalizing and adopting a revised version. In the interim, the extant “ICMJE Form for the Disclosure of Potential Conflicts of Interest” will remain in use and available as a downloadable PDF at our web site.

In a further step to avoid inconsistencies and omissions, and to help ease the disclosure process for authors, some journals will change the mechanism by which disclosures are collected. Authors are required to provide disclosures to multiple entities (e.g. to academic institutions, continuing education providers, guideline and other committees as well as medical journals). Disclosing information repeatedly, with varying reporting requirements, formats, and definitions, is frustrating for authors and contributes to problematic and controversial discrepancies across disclosures. The ICMJE will therefore accept disclosures from web-based repositories. These enable authors to maintain an inventory of their relationships and activities and create electronic disclosures tailored to the requirements of entities such as ICMJE, without having to reenter information repeatedly.

ICMJE will accept disclosures from repositories that meet the following criteria: collection and reporting of relationships and activities consistent with ICMJE requirements; no fees for individuals to enter, store, or export their data; provision of disclosures to journals electronically as well as an option for journals without a digital interface; and compliant with the General Data Protection Regulation (GDPR).

One currently available repository that is consistent with these criteria is Convey (www.convey.org), but we encourage the development of other repositories as necessary to meet regional, linguistic, and regulatory needs. A template that enables authors to create disclosures that emulate the extant “ICMJE Form for the Disclosure of Potential Conflicts of Interest” is already available at the Convey platform, and some of our journals have begun to collect author disclosures electronically in this way. This template will be updated to conform to the new ICMJE Disclosure Form when it is finalized, and all ICMJE journals can begin accepting disclosures in this manner. Ultimately, the currently employed PDF-based ICMJE form will be unavailable.

While no approach to disclosure will be perfect or foolproof, we hope the changes we propose will help promote transparency and trust. We look forward to your feedback.
